# Morphological and biochemical characterization of the cutaneous poison glands in toads (*Rhinella marina* group) from different environments

**DOI:** 10.1186/s12983-018-0294-5

**Published:** 2018-11-23

**Authors:** Pedro Luiz Mailho-Fontana, Marta Maria Antoniazzi, Juliana Mozer Sciani, Daniel Carvalho Pimenta, Katia Cristina Barbaro, Carlos Jared

**Affiliations:** 1 Laboratory of Cell Biology, Instituto Butantan, Av. Vital Brasil 1500, São Paulo, 05503-000 Brazil; 2 Laboratory of Biochemistry and Biophysics, Instituto Butantan, São Paulo, Brazil; 3 Laboratory of Immunopathology, Instituto Butantan, São Paulo, Brazil

**Keywords:** Amphibia, Bufonidae, *Rhinella*, Granular glands, Macroglands

## Abstract

**Background:**

Amphibian defence against predators and microorganisms is directly related to cutaneous glands that produce a huge number of different toxins. These glands are distributed throughout the body but can form accumulations in specific regions. When grouped in low numbers, poison glands form structures similar to warts, quite common in the dorsal skin of bufonids (toads). When accumulated in large numbers, the glands constitute protuberant structures known as macroglands, among which the parotoids are the most common ones. This work aimed at the morphological and biochemical characterization of the poison glands composing different glandular accumulations in four species of toads belonging to group *Rhinella marina* (*R. icterica*, *R. marina*, *R. schneideri* and *R. jimi*). These species constitute a good model since they possess other glandular accumulations together with the dorsal warts and the parotoids and inhabit environments with different degrees of water availability.

**Results:**

We have observed that the toads skin has three types of poison glands that can be differentiated from each other through the morphology and the chemical content of their secretion product. The distribution of these different glands throughout the body is peculiar to each toad species, except for the parotoids and the other macroglands, which are composed of an exclusive gland type that is usually different from that composing the dorsal warts. Each type of poison gland presents histochemical and biochemical peculiarities, mainly regarding protein components.

**Conclusions:**

The distribution, morphology and chemical composition of the different types of poison glands, indicate that they may have different defensive functions in each toad species.

## Introduction

One of the most striking features of amphibians is the presence of mucous and poison (or granular) glands in the skin, which play a key role in their lives, particularly in relation to chemical defence against predators and microorganisms [1, 2]. Regarding defence against predators, differently from venomous animals, in amphibians the arsenal of toxins stored in their skin cannot be inoculated in the aggressor due to the lack of an inoculatory apparatus. Poisoning only occurs when the aggressor attacks and bites the amphibian and comes into contact with the toxins via oral mucosa, which characterizes a passive mode of defence [3–6].

In venomous animals, the venom-producing glands usually have well-defined lumen, where the secretion accumulates after traversing the secretory epithelium cell membrane [7, 8]. In contrast, in anuran amphibians, the poison glands are formed by a single multinucleated cytoplasm mass, constituting a secretory syncytium [9]. There is no lumen, and the gland is totally filled with the syncytial cytoplasm matrix. The secretion, in the form of granules, after being elaborated in the syncytial periphery, is stored throughout the cytoplasm [9].

In amphibians the poison glands can be distributed singly or in the form of clusters in specific regions of the body. When aggregated in low numbers, they form structures that are similar to warts, commonly found on the dorsal skin of toads (bufonids) [10]. When grouped in large numbers, they can form conspicuous protrusions, known as macroglands [2, 10], among which the parotoids are clearly the most common: they are found in salamanders [11], phyllomedusin tree frogs [12], frogs [13] and toads [3].

Histologically, toad parotoids in genus *Rhinella* are identified as large accumulations of giant poison glands inserted into the dermis and arranged side by side, forming honeycomb-like structures [3, 6, 12]. Individual poison glands composing the parotoids show morphological characteristics that are similar to the other poison glands present in the rest of the skin. They are enveloped by a myoepithelial cell layer and are provided with a duct. However, differently from skin poison glands, the epithelium that internally lines the duct is very thick and obstructs the ductal canal, sometimes leaving just a narrow crevice in the centre [3–6, 12]. Moreover, differentiated mucous glands known as accessory glands surround each one of the ducts [3, 5, 12].

Due to the large size of the individual glands that compose the parotoids, these cutaneous organs can synthesize and store significant large amounts of poison used in chemical defence against predators [3, 5]. In toads in general, the secretion is composed of a mixture of steroids, biogenic amines and proteins, as well as mucus [3, 5, 10, 14, 15]. If ingested, toad skin secretion may cause death of several vertebrates, including humans [16–20].

Species of group *Rhinella marina* are typically large and terrestrial, with large parotoids [21]. Because they have other macroglands besides parotoids, these species constitute excellent models for macrogland morphological and biochemical studies and for the better understanding of macrogland use in passive defence. In *R. schneideri*, for example, there are conspicuous tibial (paracnemic) macroglands on the hind limbs. In *R. jimi*, besides tibial macroglands, radial macroglands are observed on the anterior limbs. Another interesting fact is the large range of environments the species of *R. marina* group inhabit: they are found in forested areas such as the Atlantic Forest (*R. icterica*) and the Amazonia (*R. marina*), in open areas as the Cerrado (*R. schneideri*), and in regions exhibiting extreme xeric conditions, such as the Brazilian semiarid Caatinga (*R. jimi*).

In relation to literature referring to toad toxinology, most investment has been given to the parotoid macroglands, especially regarding poison biochemistry. The morphology and biochemistry, as well as the functional role of the other glandular accumulations, remain practically unknown.

This paper aims at the characterization of the poison glands in *Rhinella marina, R. icterica*, *R. schneideri* and *R. jimi*. We verified that all species studied have three different types of poison glands, which are characterized by the morphology and chemical composition, besides the topographic distribution.

## Results

### Poison glands: Morphological characterization, histochemistry and distribution

**Fig. 1:**
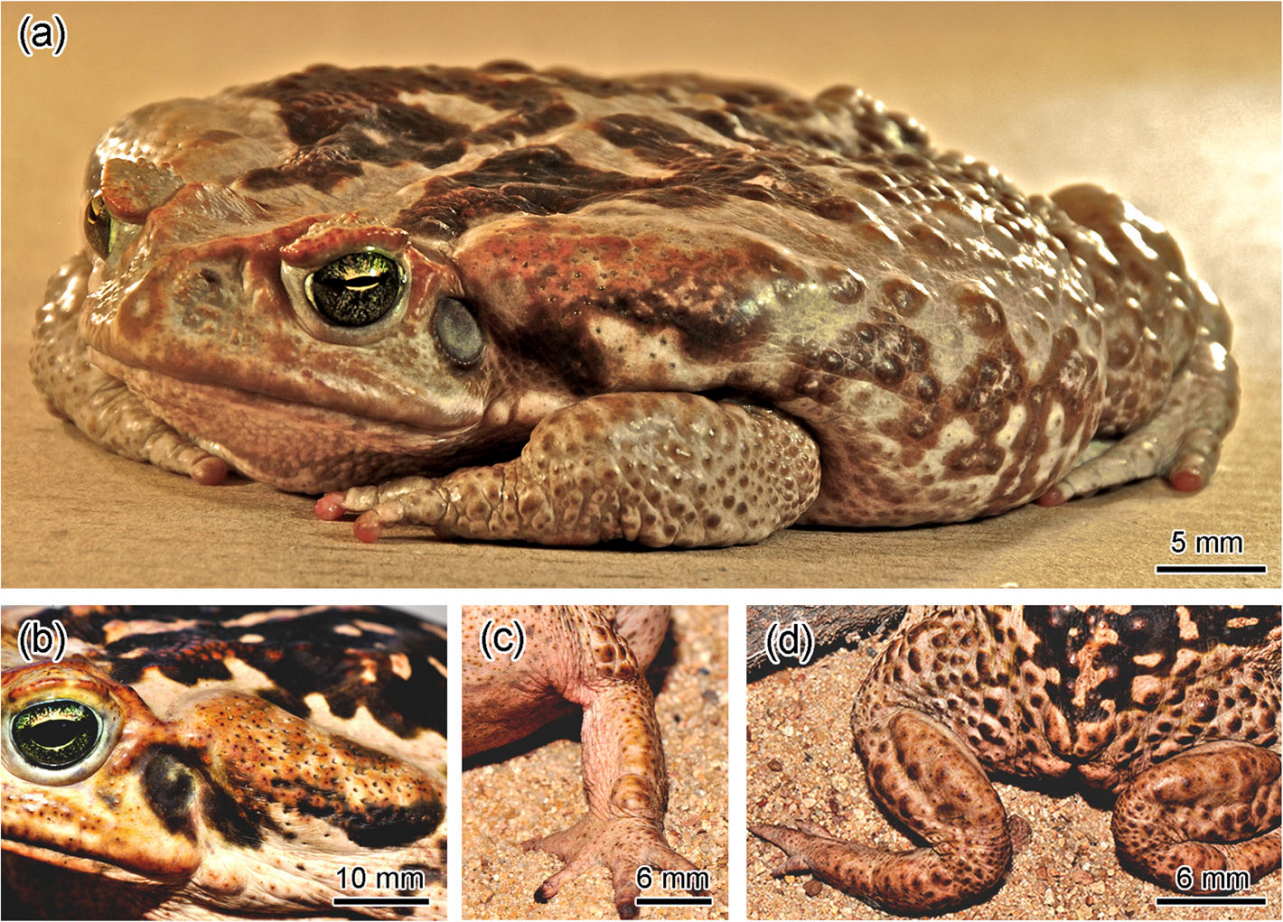
*Rhinella jimi* as a representative of the group *Rhinella marina*. (**a**) Note the parotoids, the numerous dorsal warts and the macroglands on the limbs. (**b**) A parotoid macrogland. (**c**) The radial macrogland on the forelimb. (**d**) The tibial (or paracnemic) macroglands on the hind limbs

In general, the dorsal skin surface of all studied species shows an irregular structure, with an abundance of warts (Fig. 1a). Parotoids and other macroglands are clearly distinguished from the rest of the body and from the dorsal warts due to their large volume and anatomical position (Fig. 1a–d).

**Fig. 2:**
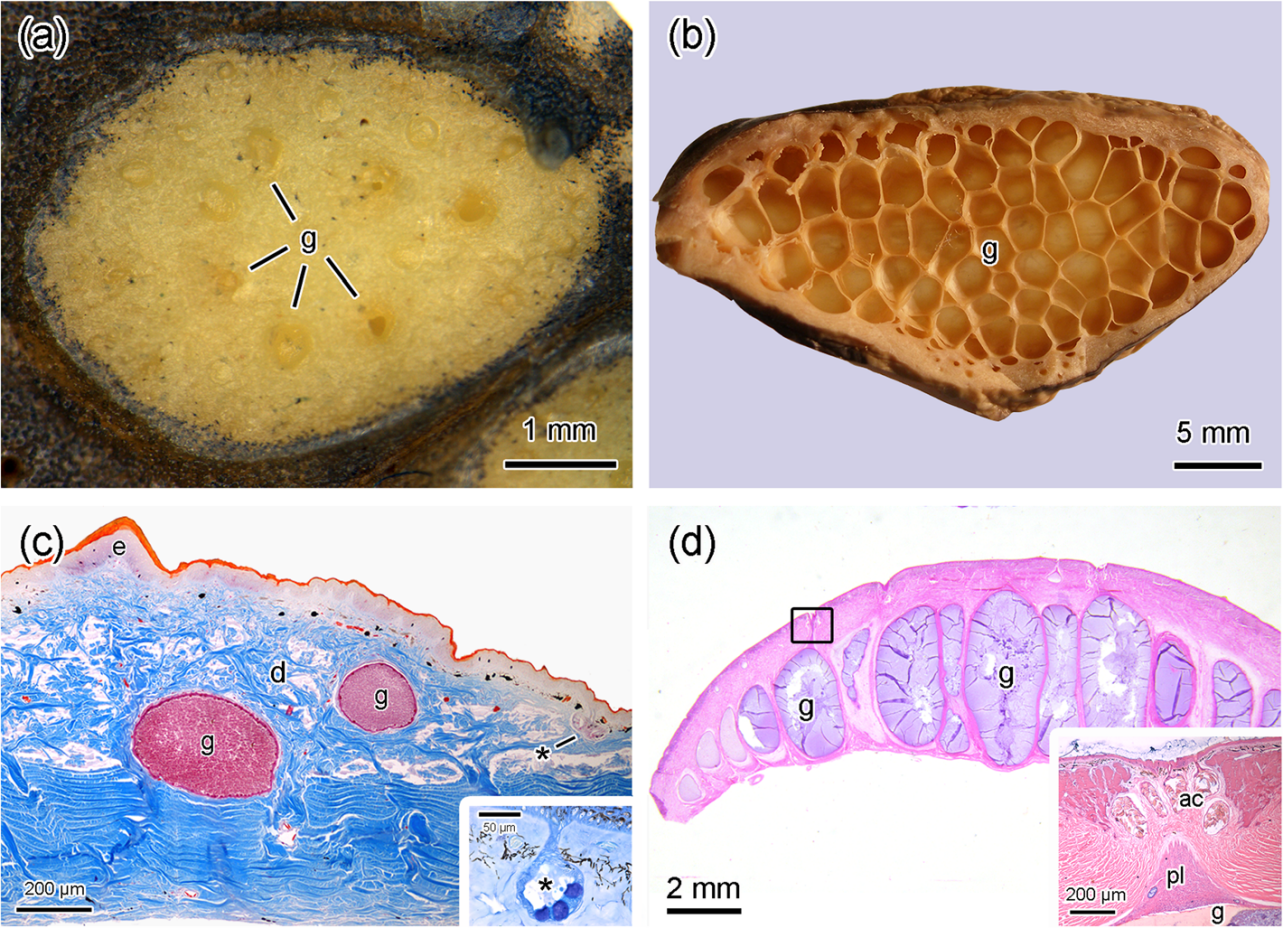
Morphological comparison between the dorsal warts and the parotoid macroglands. (**a**) Longitudinal section of a dorsal wart. Note the small number of poison glands (g). (**b**) Longitudinal section of a parotoid. The honeycomb-like structure is formed by a large number of subunits side by side, each one lodging a poison gland (g). (**c**) Transverse histological section of a dorsal wart, showing poison glands (g) and a mucous gland (*) highlighted in the insert. e, epidermis, d, dermis. (**d**) Transverse histological section of a parotoid macrogland showing the juxtaposed distribution of the poison glands (g). Note that the glands are larger in the centre of the structure and decrease towards periphery. The insert represents a high magnification of the area delimited by the rectangle, showing a duct obstructed by an epithelial plug (pl) surrounded by accessory glands (ac). Species: *Rhinella marina* (**a**, **c**, **d**) and *Rhinella icterica* (**b**). Staining: Mallory’s trichrome (**c**), toluidine blue-fuchsine (c, insert), haematoxylin-eosin (**d**)

Macroscopically, longitudinal sections across the dorsal warts reveal structures consisting of the accumulation of small poison glands (Fig. 2a). In contrast, the internal longitudinal view of the parotoids show the characteristic honeycomb structure formed by many secretory units, each one of them housing a large poison gland (Fig. 2b).

Histologically, the dorsal warts are composed of poison glands of various sizes inserted into the dermis and completely filled with secretion (Fig. 2c). Mucous glands consisting of a monolayer of secretory cells organized around a characteristic central lumen (Fig. 2c insert), are also observed just below the epidermis.

In the species here studied, the parotoids present a general morphology much similar to that described for all other species of *Rhinella marina* group [3, 5, 6, 22]: they are basically composed of very elongated poison glands that decrease in diameter from the macroglandular centre towards the periphery (Fig. 2d). Each of these giant poison glands is provided with a single duct obstructed by a plug composed of epithelial tissue (Fig. 2d inserted). Each duct is surrounded by a number of accessory glands, which are differentiated mucous glands (Fig. 2d inserted).

In the case of *Rhinella jimi* (Fig. 1a), it was clearly observed that, besides the parotoids (Fig. 1b), they show radial (Fig. 1c) and tibial macroglands (Fig. 1d), all of them with very similar morphology, although with variations in size and shape.

When the general topology, morphology and histochemistry of the granular glands are analysed together with the morphological appearance of the poison granules, it is possible to classify these glands into three basic types: skin common poison glands (COM), parotoid poison glands (PAR) and peripheral parotoid poison glands (PER).

**Fig. 3:**
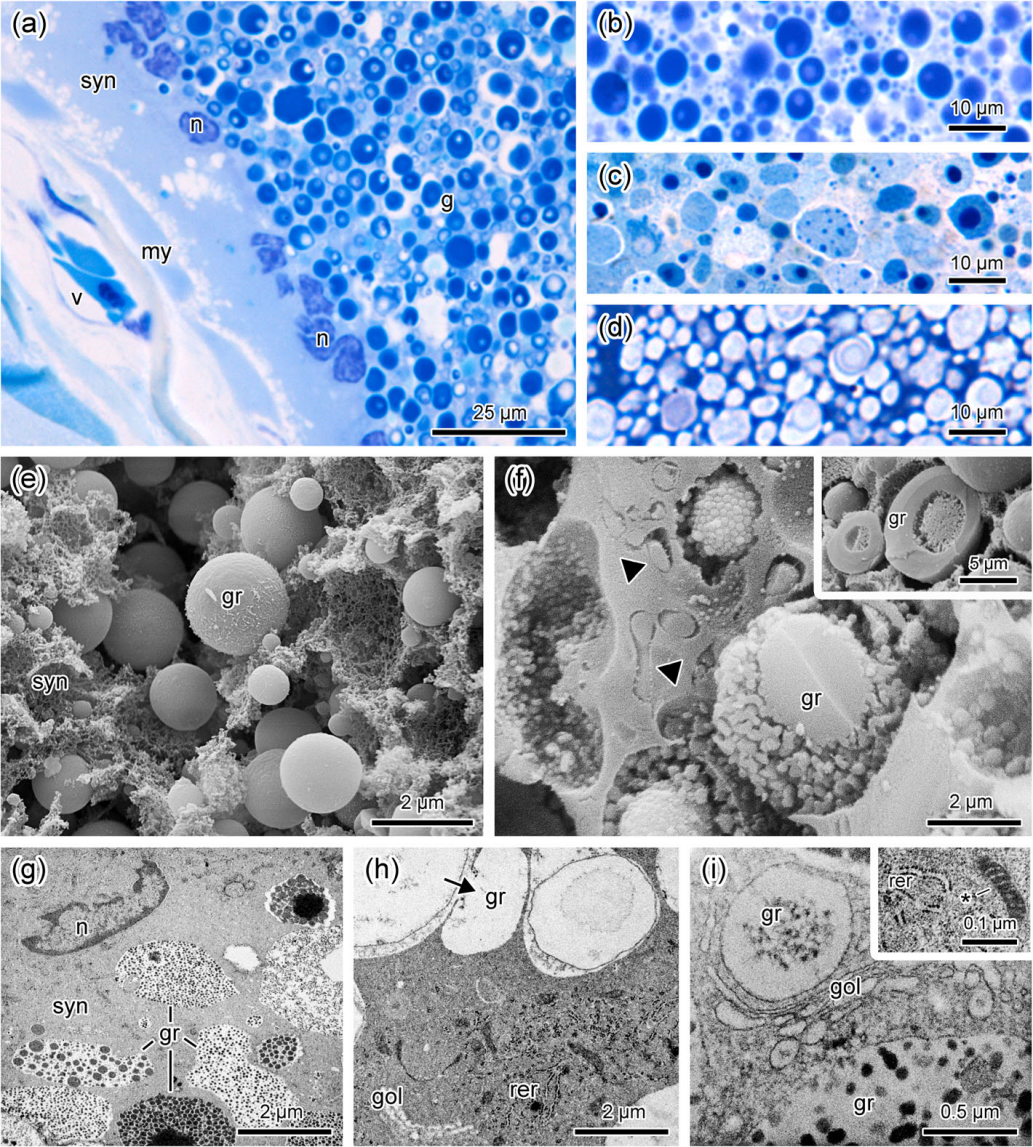
Morphological characteristics of poison glands of type COM, typical of dorsal warts. (**a**) Part of a syncytium (syn) with peripheral nuclei (n) and a large amount of heterogeneous poison granules (**g**). my, myoepithelial layer, v, blood vessel. (**b**-**d**) In all studied species, the poison granules show internal subunits. (**e**) Poison granules (gr) with smooth surface immersed in the syncytial cytoplasm matrix (syn). (**f**) Syncytial granules (gr) with rough surface and different degrees of compaction, and lamellae with small granules inside (arrowheads). Insert: granules (gr) with compact periphery and internal subunits. (**g**) Ultrathin section evidencing heterogeneity of the poison granules (gr). n, nucleus; syn, syncytial cytoplasm. (**h** and **i**) Syncytial organelles amongst poison granules (gr). Notice the fusion between two poison granules (arrow). gol, Golgi apparatus; rer, rough endoplasmic reticulum; *, mitochondrium. Species: *Rhinella icterica* (**a**, **e**), *Rhinella schneideri* (**b**, **f** insert), *Rhinella marina* (**c**, **f**, **g**) *Rhinella jimi* (**d**, **h**, **i**). Histological sections stained with toluidine blue-fuchsine (**a**-**d**). SEM cryofractures (**e**-**f**). TEM (**g**-**i**)

Since COM poison glands are ordinary components of the dorsal skin, including the dorsal warts, and are also found throughout the ventral and pelvic skin, they are much more abundant when compared with the other two poison gland types. In COM glands the syncytial cytoplasm is light and have numerous evident peripheral nuclei and the poison granules are typically spherical, heterogeneously staining with toluidine blue (Fig. 3a–d). Although each species has its own typical characteristics, the general morphology of the secretion granules follows a similar pattern, always presenting internal subunits, either pale or dense (Fig. 3a–d). Cryofractures observed by SEM reveal that the surface of the granules is usually smooth (Fig. 3e) although it may show a rough appearance, depending on the species (Fig. 3f). The fractured granules exhibit subunits with a wide variety of shapes and different degrees of compaction (Fig. 3f insert). These subunits correspond to the unidimensional structures observed in the histological sections (Fig. 3a–d).

Analysis by TEM shows that the syncytium of COM glands is quite electron lucent and forms a halo around each one of the granules (Fig. 3g and h). The shape, size and electron density of the granules are variable, depending on the species (Fig. 3g–i). Organelles involved in poison synthesis such as rough endoplasmic reticulum and Golgi apparatus (Fig. 3h and i) are observed among the granules, throughout the syncytium, even in the most central portion of the gland.

**Fig. 4:**
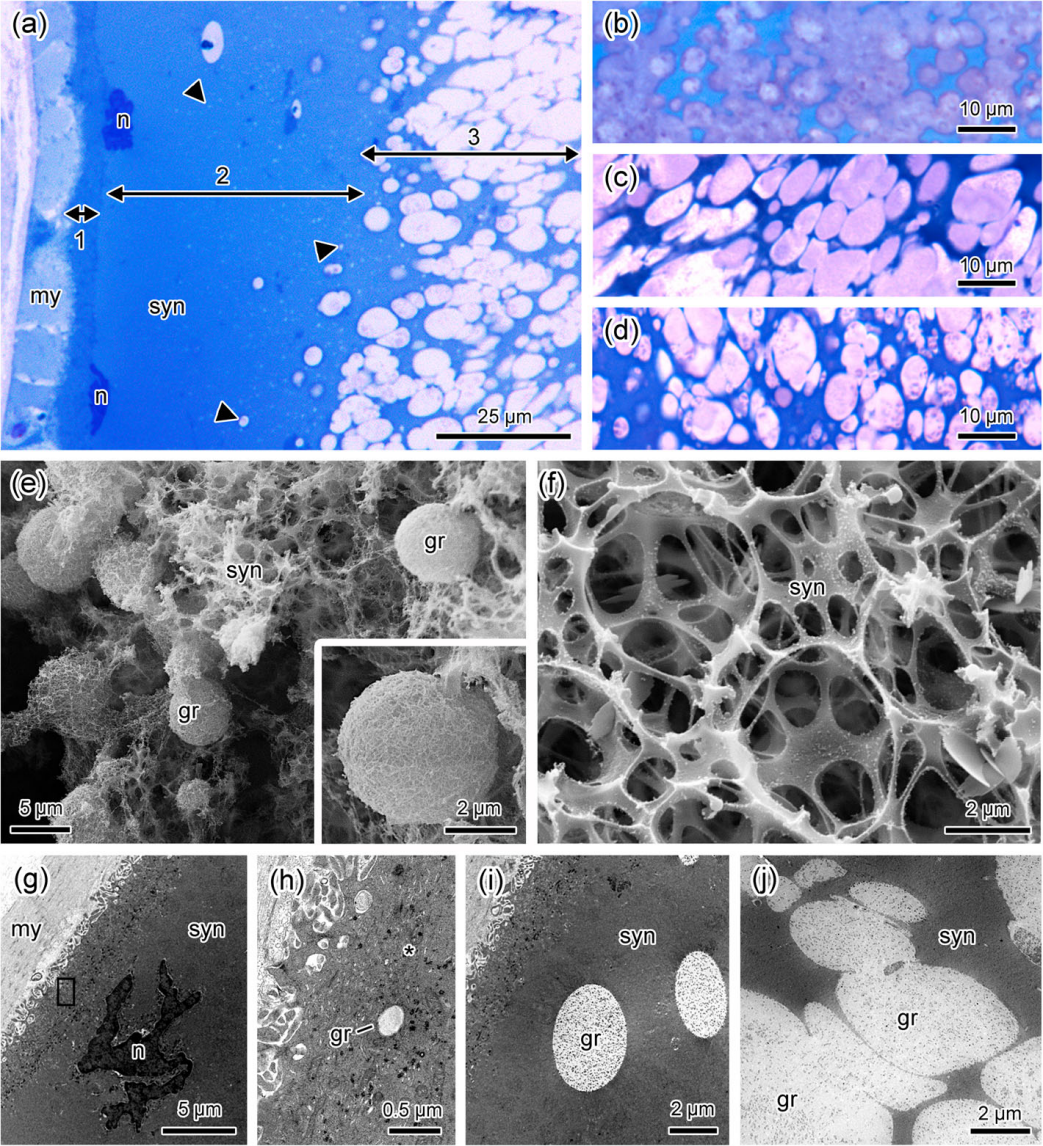
Morphological characteristics of poison glands of type PAR, typical of parotoids. (**a**) Part of the syncytium (syn) with low number of nuclei (n) and the typical arrangement in layers. The first more external thin layer (1) is followed by the second intermediate layer (2) containing the nuclei (n) and small poison granules of different sizes (arrowheads). The third inner layer (3) is characterized by the high number of poison granules of different sizes and shapes, without internal subunits, many of them fusing to each other (**b**-**d**). (**e**) Part of the inner layer showing the poison granules (gr) immersed in the syncytial cytoplasm matrix (syn). Insert: higher magnification of a granule resembling a wool ball. (**f**) The syncytial cytoplasm matrix (syn) with spongy appearance, after removal of the granules. (**g**) Ultrastructure of syncytial layers 1 and 2. my, myoepithelium; n, nucleus; sy, syncytium. (**h**) High magnification of the region marked by the rectangle in (**g**). Layer 1 contains high numbers of organelles (*) such as endoplasmic reticulum, mitochondria and Golgi apparatus. (**i**) Syncytial layer 2, is devoid of organelles but show small electron lucent poison granules (gr). syn, syncytium. (**j**) Syncytial layer 3 is full of fusing electron lucent poison granules (gr), without internal subunits. Species: *Rhinella icterica* (**a**, **e**, **i**), *Rhinella schneideri* (**b**), *Rhinella marina* (**c**, **g**, **h**, **j**) and *Rhinella jimi* (**d**, **f**). Histological sections stained with toluidine blue-fuchsine (**a**-**d**). SEM cryofractures (**e**-**f**). TEM (**g**-**j**)

Poison glands of PAR type are the major component of the parotoids in all studied species. Specifically, in *Rhinella schneideri* and *R. jimi*, the other macroglands besides the parotoids are also composed by PAR glands. Histologically, PAR glands consist on a dense syncytium with a lower number of nuclei when compared to COM glands (Fig. 4a). Moreover, the syncytium of PAR glands is characterized by a peripheral region composed of three clearly distinguishable layers (Fig. 4a). The first more external layer, subjacent to the glandular myoepithelium, is dense and about 2 μm thick and internally extends to the region where the syncytial nuclei are located (Fig. 4a). The second more internal layer, dense, wide and smooth, is characterized by scattered poison granules at different stages of formation and with a variety of sizes (Fig. 4a). The third central portion of the syncytium is full of light granules, most of them fusing to each other, immersed in a cytoplasm matrix that strongly stains with toluidine blue (Fig. 4a). Regardless the species analysed, the granules in PAR glands are always spherical or elliptical, very heterogeneous in size, and stain light and homogeneously with toluidine blue (Fig. 4a–d). Cryofractures observed by SEM reveal the wool-ball aspect of the granules (Fig. 4e) and clearly show that they are lodged within the cytoplasm matrix, which confers to the whole structure a spongy appearance (Fig. 4f).

Differently from COM glands, TEM observation of PAR glands reveals that the first external layer of the syncytium concentrates, besides the nuclei, all cytoplasm organelles (Fig. 4g–i), such as mitochondria, smooth and rough endoplasmic reticulum and Golgi apparatus (Fig. 4h). These organelles, in the second syncytial layer, give rise to the secretion granules (Fig. 4i), which gradually fuse to each other, increasing in size while they migrate towards the third central layer (Fig. 4j). The poison granules of PAR glands are quite homogeneous, and their electron lucent content do not outstand much from the syncytial matrix (Fig. 4j).

**Fig. 5:**
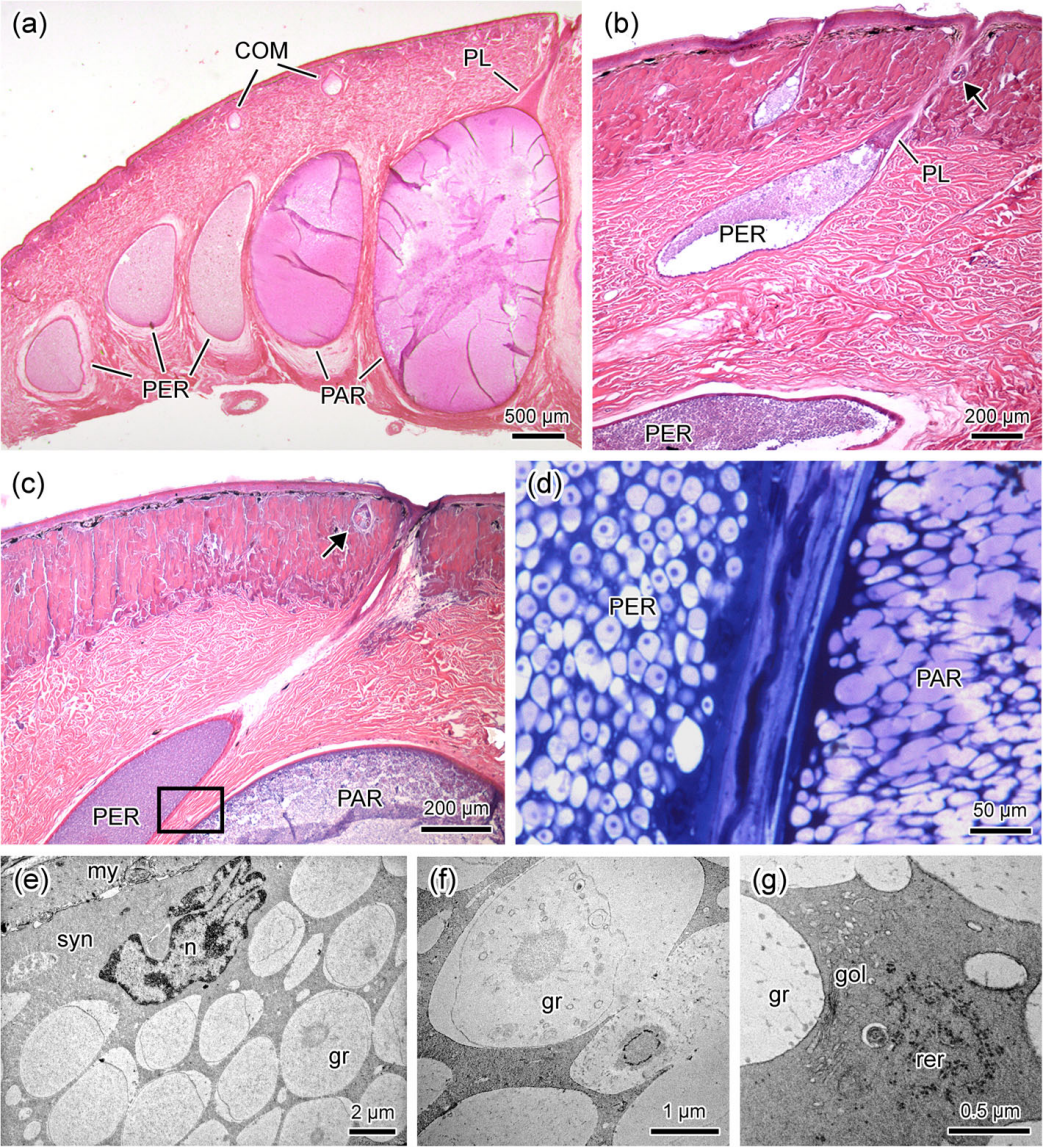
Morphological characteristics of poison glands of type PER, typical from the margins of the parotoids and other macroglands. (**a**) Note that in the parotoid all described glandular types (COM, PAR and PER) are present. (**b**) High magnification of the parotoid margin showing PER poison glands at different stages of development. Note that one of these glands shows an epithelial plug (PL). A mucous gland is seen on the duct side (arrow). (**c**) Margin of the parotoid where a PER gland is observed on the side of a PAR gland. Note again a mucous gland (arrow) next to the glandular duct. (**d**) High magnification of a region equivalent to that marked by the rectangle in the previous image. Note the difference between the granules of PER and PAR glands. PER glands have subunits, while PAR glands are light and homogeneous and fuse to each other. (**e**) Differently from PAR glands, PER glands do not present polarization of cytoplasm organelles. gr, granules; my, myoepithelial layer; n, nucleus; syn, syncytium. (**f**) Poison granules (gr) have internal subunits. (**g**) Cytoplasm organelles disperse amongst poison granules (gr). gol, Golgi apparatus, rer, rough endoplasmic reticulum. Species: *Rhinella marina* (**a**-**d**), *Rhinella icterica* (**e**-**g**). Histological sections (a-d). Staining: haematoxylin-eosin (**a**-**c**), toluidine blue-fuchsine (**d**). TEM (**e**-**g**)

A third type of poison gland, here named PER, was found exclusively at the periphery (or margins) of the parotoids, as well as of other macroglands (Fig. 5a). PER glands are of an intermediate size between PAR and COM glands and, similarly to PAR glands, their syncytium is dense, and their ducts are always surrounded by accessory glands (Fig. 5b and c). At the same time, similarly to COM glands, PER glands histology and ultrastructure (TEM) reveal the conspicuous syncytial nuclei and the secretion granules that are spherical and with internal subunits (Fig. 5d–f). Moreover, organelles are dispersed amongst secretion granules throughout the syncytial cytoplasm is also very similar to what is observed in COM glands (Fig. 5g).

**Fig. 6:**
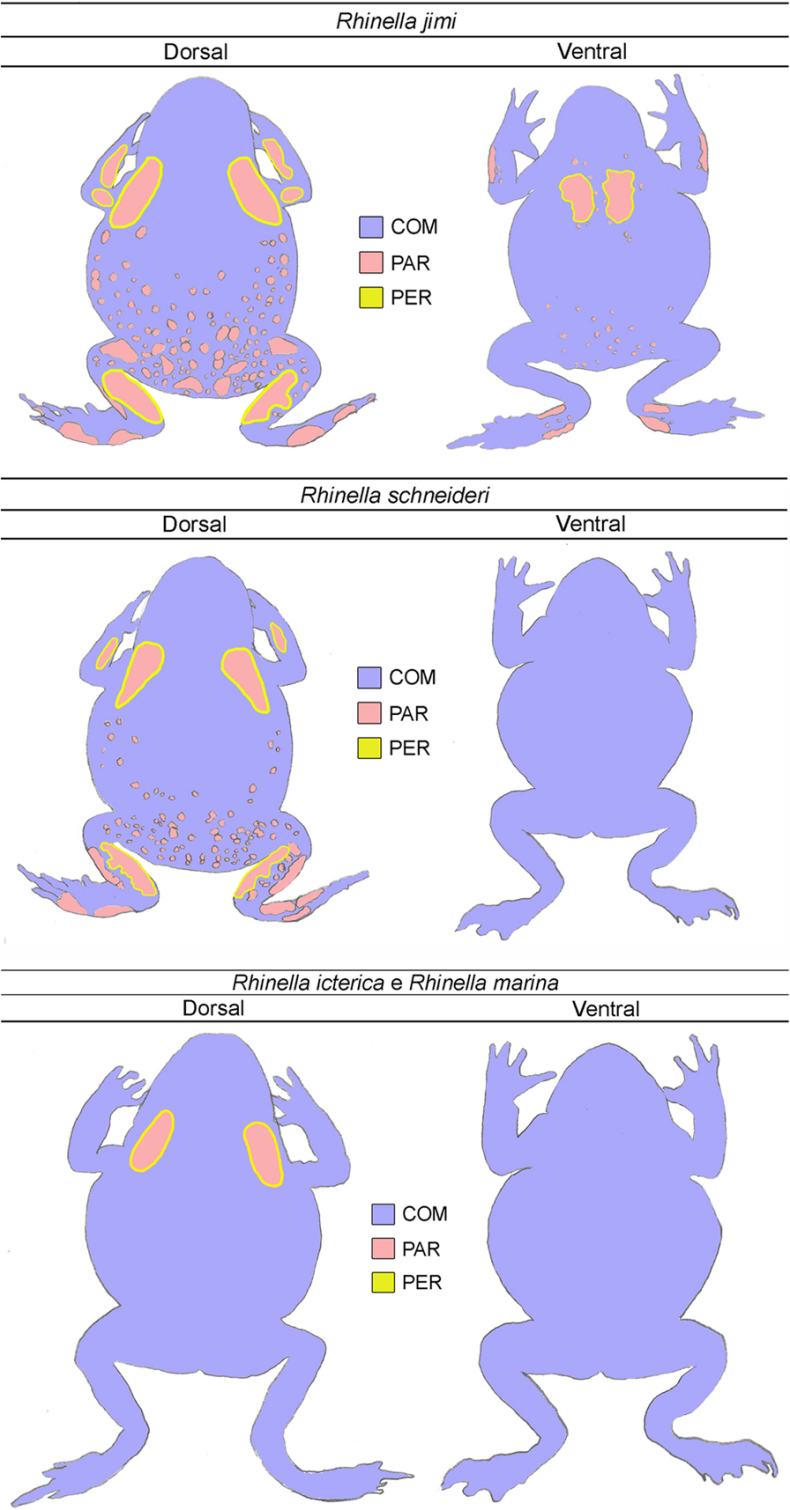
Body distribution of COM, PAR and PER poison glands in the four studied toads. Note that only in *Rhinella schneideri* and *Rhinella jimi* poison glands of type PAR are also present disperse on the dorsal skin, forming large numbers of aggregates, mainly in *R. jimi*. In this latter species PAR glands are also present on the ventral face, forming large accumulations on the pectoral region. Schemes were drawn from the observation of entire skins removed from the animals and analysed from the inner face

When the internal side of the skin preserved in formalin is observed with the naked eye, the glands of type PAR stand out due to their brownish colour. The use of this simple methodology made possible the observation that, exclusively in *Rhinella schneideri* and *R. jimi*, PAR glands, besides their occurrence in the macroglands, they are also present spread on other regions of the body in association with COM glands, especially in the dorsal skin, where they occur in large numbers, although with a smaller size when compared to those in the parotoids and other macroglands (Fig. 6). Subsequently, the identification of PAR glands was confirmed through histological and histochemical analysis (data not shown). In *R. schneideri*, PAR glands are found exclusively on the dorsal skin, occupying around 20% of the total area of the back (Fig. 6). In contrast, in *R. jimi*, these glands are even more widespread throughout the body, reaching 25% of the dorsal skin and occupying 6% of the ventral skin, where they form a non-protuberant occult glandular accumulation in the pectoral region (Fig. 6). The distribution pattern of PAR glands indicates that they are more abundant in the extremities and sides of the body (Fig. 6).

**Fig. 7:**
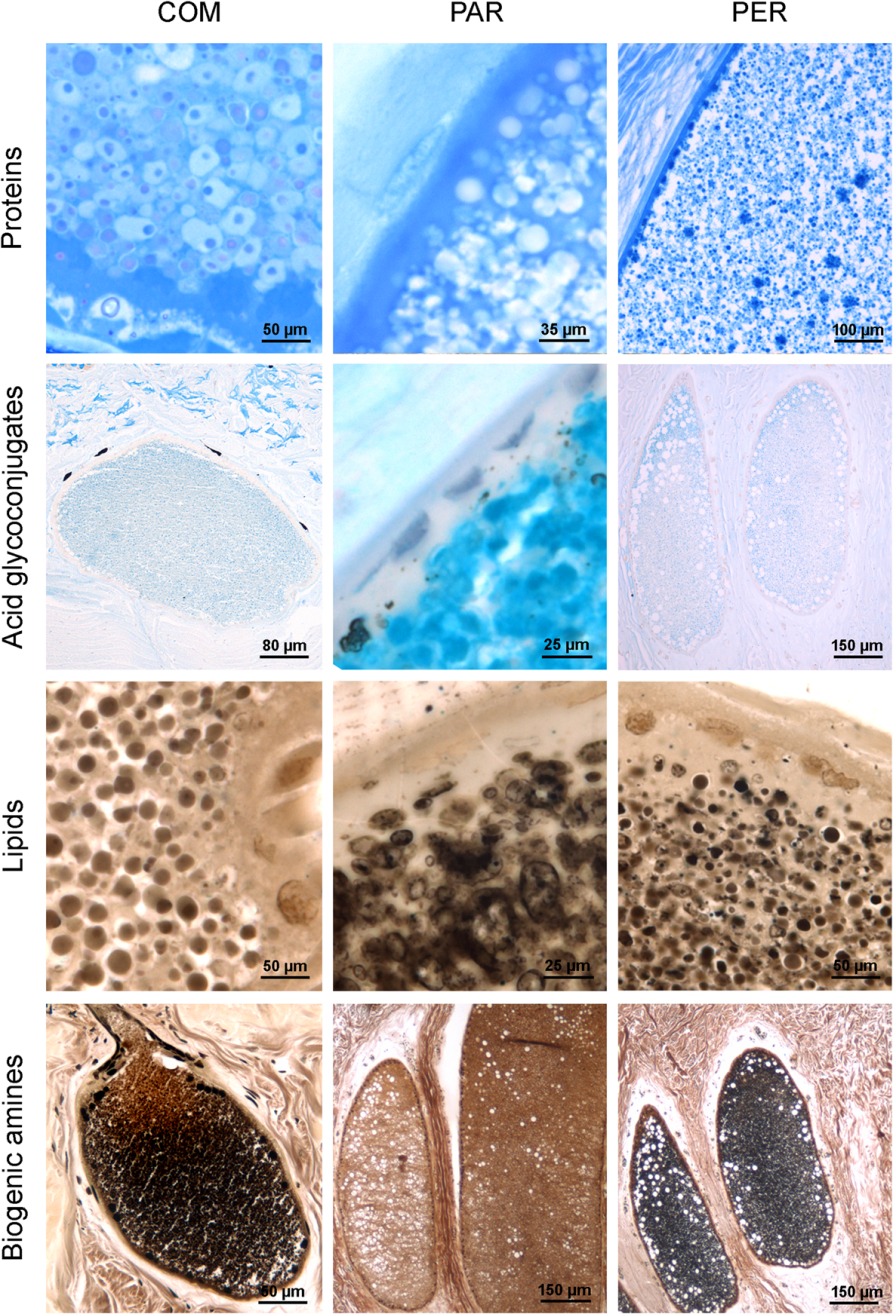
Histochemical composition of COM, PAR and PER poison glands. Regardless of the species studied, all glands secrete proteins, acid glycoconjugates (mainly PAR glands), lipids and biogenic amines. Note the similarity in composition of the secretion in COM and PER glands. Species: *Rhinella marina* (first and third rows) and *Rhinella icterica* (second and fourth rows). Histochemical methods: bromophenol blue (first row), alcian blue pH 2.5 (second row), Sudan black (third row) and modified Masson-Fontana (fourth row)

The glands of types COM and PER are histochemically different from PAR glands since their poison granules avidly stain with bromophenol blue, indicating high protein content (Fig. 7). On the other hand, the granules of PAR glands are rich in acid glycoconjugates, since they are strongly positive to alcian blue pH 2.5 (Fig. 7), but are immersed in a bromophenol blue positive matrix, rich in protein content. All three types of gland similarly react to the modified Masson-Fontana and Sudan black methods, indicating, respectively, the presence of biogenic amines and lipids in their contents (Fig. 7).

Table 1 summarizes the main morphological and histochemical results obtained for COM, PAR and PER glands.

**Table 1:** Main morphological and histochemical characteristics of the three types of poison glands (COM, PAR and PER)

	COM	PAR	PER
Topological distribution	Typical of dorsal warts. Present in other body regions in all species	Typical of macroglands. Exclusively in *Rs* e *Rj*, in dorsal warts and other body regions	Exclusive at the periphery of macroglands
Morphological characterization	Spherical granules with heterogeneous subunits. Syncytium with low density and many conspicuous nuclei	Elliptic and homogeneous granules, without internal subunits. Syncytium with high density and low number of nuclei	Elliptic or spherical granules, with internal subunits. Dense syncytium with conspicuous nuclei
Histochemical characterization of granules	Positive to proteins, lipids and biogenic amines	Positive to acid glycoconjugates, lipids and biogenic amines	Positive to proteins, lipids and biogenic amines

### Biochemical characterization of the poison in the dorsal warts and in the macroglands

**Fig. 8:**
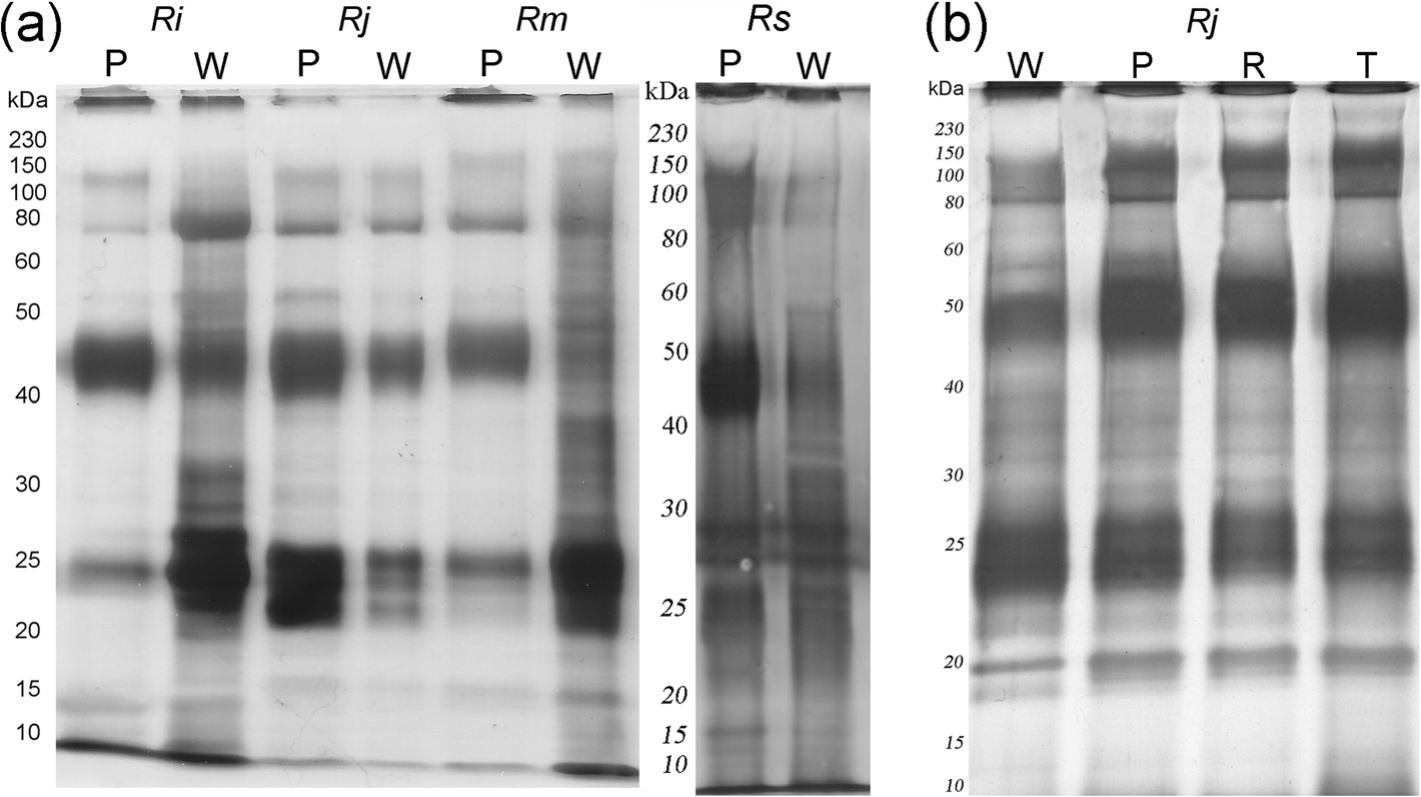
SDS-PAGE comparison of the poison extracted from dorsal warts with that extracted from parotoids and other macroglands in the four species studied. (**a**) Electrophoretic profiles of the dorsal warts poison (W) and the parotoid poison (P). Note that the diversity of bands in W is much larger when compared to that of P. (**b**) Electrophoretic profiles of the poisons of dorsal warts (W) parotoids (P), radial macroglands (R) and tibial macroglands (T) in *Rhinella jimi*. Note the similarity of band pattern for all types of glandular accumulations in this species. Legends: *Rhinella icterica* (*Ri*), *Rhinella jimi* (*Rj*), *Rhinella marina* (*Rm*) and *Rhinella schneideri* (*Rs*)

SDS-PAGE of the poison extracted from the dorsal warts, structures that in *Rhinella icterica* and *R. marina* are typically composed of COM glands, presents a larger diversity of bands when compared to the poison extracted from the parotoids that are basically composed of PAR glands (Fig. 8a). In *R. jimi*, the poison from the dorsal warts, structures composed of PAR glands (differently from the other three toad species), presents a SDS-PAGE profile very similar to the profile observed for the poison extracted from parotoid, tibial and radial macroglands (Fig. 8b), structures composed of PAR glands in the four studied species.

**Fig. 9:**
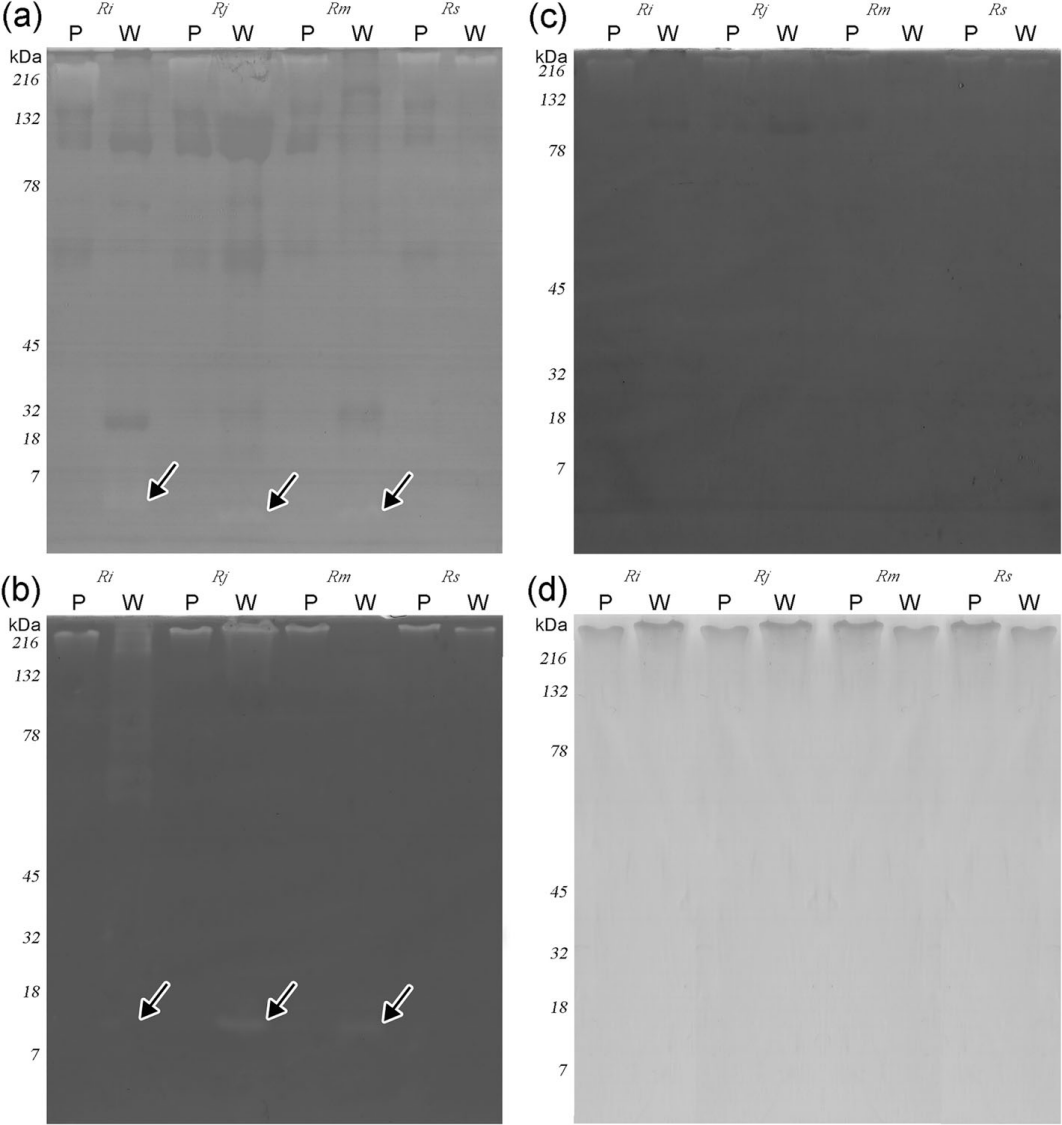
Profiles of enzymatic activity of poisons from dorsal warts (W) and parotoids (P) in the four species studied. (**a**) gelatinolytic activity. (**b**) fibrinogenolytic activity, (**c**) caseinolytic activity and (**d**) hyaluronidase activity. Activity was detected against gelatine (**a**) and fibrinogen (**b**) and is present only in the poison extracted from dorsal warts (arrows). Legends: *Rhinella icterica* (*Ri*), *Rhinella jimi* (*Rj*), *Rhinella marina* (*Rm*) and *Rhinella schneideri* (*Rs*)

In all four toad species, the poison from the parotoids was not able to cleave the tested substrates. In contrast, the poison extracted from the dorsal warts showed some bands with low proteolytic activity (Fig. 9). When gelatine was used as substrate in the gels, the poisons of *Rhinella icterica*, *R. jimi* and *R. marina* formed bands at approximately 5 kDa (Fig. 9a). In the gel containing fibrinogen, the poison of the dorsal warts of *R. jimi* and *R. marina* presented bands around 10 kDa (Fig. 9b). None of the tested samples showed caseinolytic or hyaluronidase activities (Fig. 9c and d).

**Fig. 10:**
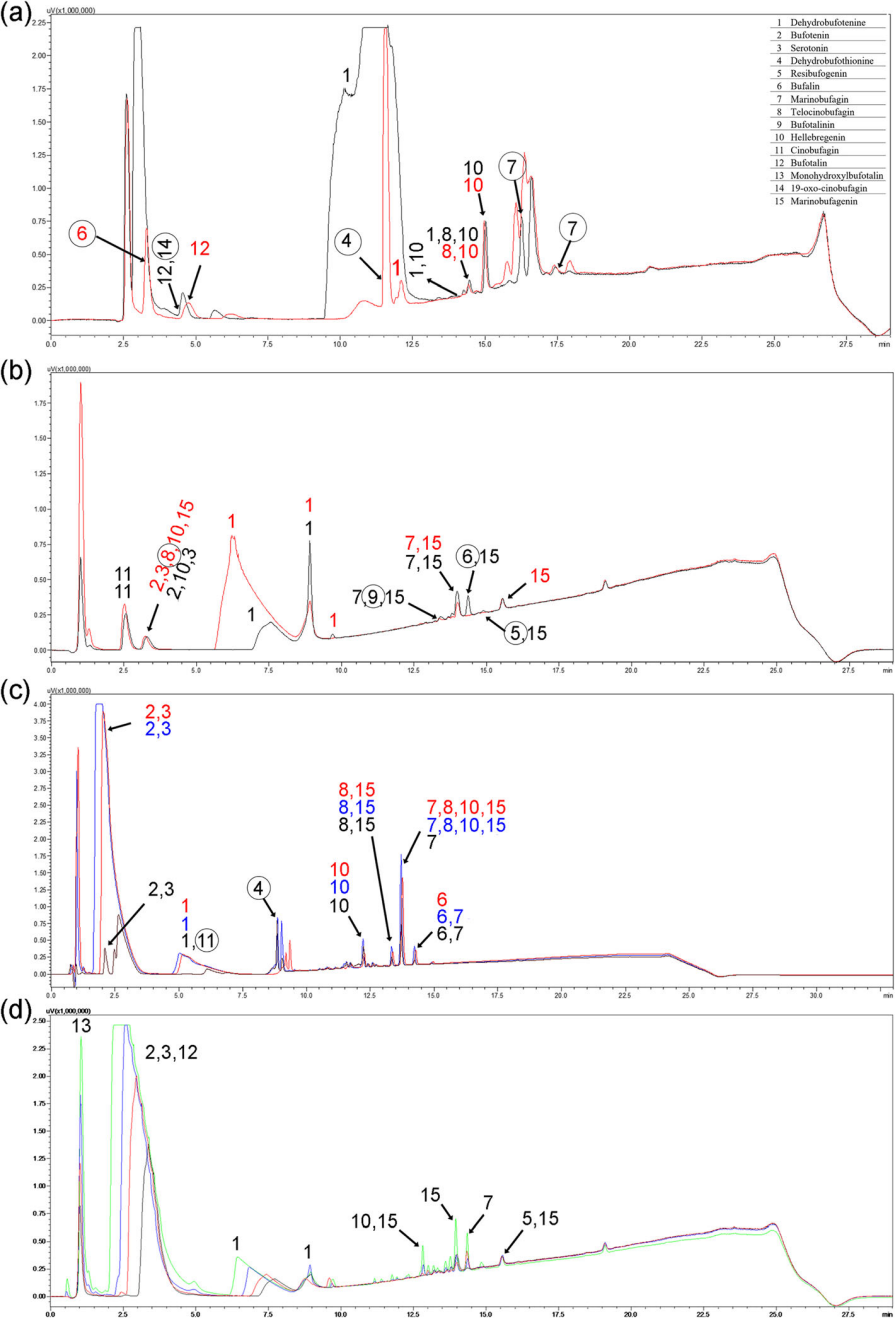
Profile C18-RP-HPLC (λ = 214 nm) of poison extracted from different glandular accumulations. (**a**) *Rhinella icterica*. Dorsal warts (red) and parotoids (black). (**b**) *Rhinella marina*. Dorsal warts (red) and parotoids (black). (**c**) *Rhinella schneideri*. Dorsal warts (red), parotoids (black) and tibial (paracnemic) macroglands (blue). (**d**) *Rhinella jimi*. Dorsal warts (red), parotoids (black), tibial (paracnemic) macroglands (blue) and radial macroglands (green). The numbers refer to the molecules identified in the poisons. Circles around the numbers correspond to exclusive molecules in each poison. Note that only in *R. jimi* the chromatographic profiles are similar for all types of glandular accumulations indicating the same poison composition

The analysis of the poisons of the four species by RP-HPLC and mass spectrometry, revealed that, besides proteins, they are composed of low molecular mass molecules, such as biogenic amines, which are eluted at the beginning of the chromatography, and steroids, which are eluted in later times. Poisons of the dorsal warts and of the macroglands have very similar low molecular components, although with quantitative differences, in addition to some other exclusive molecules (Fig. 10a–d). In *R. icterica*, *R. marina* and *R. schneideri*, the poison of the dorsal warts shows different molecules from that of the parotoids (and, in the case *R. schneideri*, tibial macroglands also) (Fig. 10a–c). Exclusively in *R. jimi*, the poison of the dorsal warts is very similar to that of the parotoid, radial and tibial macroglands (Fig. 10d), which is compatible with the similarities observed through morphology, histochemistry and SDS-PAGE.

## Discussion

Although many papers have described the morphology of amphibian poison glands since the nineteenth century [23–26], none of them invested in an integrative approach, trying to gather morphological data with the amphibian biology and natural history [13]. More recently, some studies have examined these glands in an interdisciplinary context, in order to increase the understanding of the morphology, biochemistry, and functioning of these structures [5, 6, 12, 13, 15, 22, 27–30].

Particularly within bufonids, the parotoid macroglands were always the preferred target of study, probably due to their more conspicuous structure. Very little attention has been given to other glandular accumulations such as the dorsal warts or the tibial and radial macroglands regarding the morphology and the biochemistry of their secretion. Our integrative approach, taking into consideration all types of glandular accumulation throughout the body, clearly revealed the existence of different types of poison glands (here named COM, PAR and PER glands) with peculiar distribution in each species.

In relation to glandular distribution, glands of type PAR showed a striking irregular pattern. While in *Rhinella icterica* and *R. marina* PAR glands are exclusively found composing the parotoids, in *R. schneideri* and *R. jimi*, besides composing the tibial and radial macroglands, PAR glands are also found in dorsal warts, as well as in other regions of the body. In *R. jimi*, for example, there is an evident accumulation of this gland type in the pectoral region, but, contrasting with the protuberant parotoids and warts on the back, they developed inward, not displaying any external volume. The development of macroglands, as well as their tendency to spread throughout the body, seems to provide to *R. schneideri* and *R. jimi* a greater protection against predators through the use of chemical defence when compared to the other toad species. Additionally, the macroglands may also provide physical protection to the regions where they are located, in a function similar to that of the parotoids, serving as bullet-proof vests and absorbing the bite impact, as described by Regis-Alves et al. [6].

Due to the unique morphological peculiarities presented by the three different glandular types, and to their pattern of distribution in each species, it seems unlikely that these glands could simply represent different stages of maturation of a single glandular type. Some studies have indicated that the macroglands are, in several anuran families, composed of glands with a different morphology from those found in the rest of the skin [12, 27, 31]. Nevertheless, none of the studies proceeded a comparative biochemistry of the poison of these two gland types. In this sense, Mailho-Fontana et al. [13] represented a breakthrough, showing that the poison of different types of macroglands in the frog *Odontophrynus cultripes* have different chemical compositions. The authors, however, did not discriminate the chemical composition of the poison from the dorsal warts.

The evolutionary origin of amphibian parotoids and other skin macroglands remains completely unknown. It is assumed that these glandular accumulations may have originated from the common skin poison glands as a response to selective pressure imposed by predation [10]. Thus, body areas most likely to be attacked by predators, such as the extremities and flanks, must have developed differentiated glands in the skin. In this context, PER glands, which are specifically present at the periphery of the macroglands, seem to give an important clue about the process of formation of glandular accumulations. Our data indicate that PER glands may represent intermediate stages of glandular development, since they display hybrid morphology, with characteristics of both COM and PAR glands. Although the differentiation process is still totally unknown, facing our results, two possibilities are raised about PER glands: they may represent different evolutionary stages or, alternatively, they may represent intermediate ontogenetic stages of COM glands towards a gradual development into PAR glands throughout the toad’s life. At first glance, the latter hypothesis seems less plausible than the first one, since the present study was based exclusively on adult animals. Moreover, other studies show that the parotoid poison granules in newly metamorphosed toads already have morphology very similar to that of adults [32].

The simultaneous analysis of glandular morphology and chemical composition indicates a clear correlation between the form of the secretion granules and the class of molecules they contain. Granules mainly consisting of protein (as in COM glands) are always dense and have internal subunits. On the other hand, granules containing lower levels of proteins (as in PAR glands), do not show internal dense forms and, on the contrary, appear homogeneous. Our biochemical data confirm the difference between the poison of dorsal warts, with a higher diversity of proteins, and that of the parotoids. Literature analysis concerning the poison of bufonid parotoids, brings evidence that proteins, despite occurring in considerable amounts in the poison, have never received as much attention as the other compounds. Studies have shown that 25 to 35% of the dry weight of toad paratoid poison is represented by proteins [14, 15]. However, the identity and actual function of this category of molecules in bufonid poison is still quite controversial. Sciani et al. [33] questioned the role of parotoid proteins in chemical defence, since these authors did not find significant differences among several species of genus *Rhinella*.

In an attempt to clarify the functionality of poison proteins extracted from dorsal warts and parotoids of the four toad species, zymographic techniques were applied on SDS-PAGE gels to evaluate the existence of proteases. Proteolysis, albeit very mildly, was the only activity we could observe in the poison of dorsal warts emphasizing once more the differences among the gland types here described. This result agrees with that was observed by Sousa-Filho et al. [15], who showed that most proteins in the poison of *Rhinella scheneideri* parotoids are constitutive, and even those still unknown proteins do not show proteolytic activity.

Toads, similarly to other amphibians, have a typical passive mode of defence and, when bitten by a predator/aggressor, are unable to trigger a poisoning system [5]. In contrast, venomous animals are able to actively inject their venom, usually rich in proteases, causing damage to the extracellular matrix and facilitating venom diffusion through the tissues [28, 34–39]. On the other hand, in toads, the steroids and biogenic amines present in the poison, due to their low molecular mass, must be able to cross the oral mucosa without the aid of proteases, rapidly reaching the bloodstream, and acting on the cardiovascular system where they increase blood pressure and/or raise heart contraction [40, 41].

The analysis of our data indicates the possibility that larger numbers of PAR poison glands on the body skin may favour toad adaptation to the terrestrial environment. This hypothesis is mainly based on the high hydrophilicity demonstrated by PAR glands, which are rich in acid glycoconjugates that may facilitate water absorption and retention during periods of drought, as discussed by Toledo & Jared [42] and later explored by Van Bocxlaer et al. [43]. This idea seems even more plausible considering the the abundance of PAR glands both in *Rhinella schneideri* and *R. jimi*, species typical of xeric biomes. Taking into consideration that the skin is the interface between the animal and the environment, this hypothesis should be better investigated through the use of other approaches, opening new perspectives of study specially in the areas of physiology and ecology.

## Conclusions

The present study clearly shows that, depending on the toad species, the poison produced by the dorsal warts is different from that produced by the macroglands. This observation opens a new perspective for future studies aiming to clarify the role of each one of these different skin gland types. Moreover, we concluded that the morphology of the poison granules is directly related to their biochemical composition. Regarding these aspects, we propose that the study of a larger number of toad species would help to elucidate the morphological and biochemical variation of bufonid skin glands, in an enlarged phylogenetic scenario. Finally, our results show that toads inhabiting xeric environments such as *Rhinella jimi* and, to a lesser extent, *R. schneideri*, have similar distribution of their skin gland types, although in different proportions. Summarizing, the data here presented indicate that the development of a more robust cutaneous defensive arsenal among toads, at least within the group *R. marina*, may have simultaneously contributed to a successful colonization of dry environments.

## Methods

### Animals

Specimens of *Rhinella icterica* and *Rhinella schneideri* were collected in different locations of São Paulo state, Brazil, in forested habitats (Atlantic Rainforest) or open savannah (Cerrado), respectively. Specimens of *Rhinella marina* were obtained in Amazonian Rainforest in Belterra, Pará. Specimens of *Rhinella jimi* were collected in Brazilian semiarid (Caatinga) in Angicos, Rio Grande do Norte state. Animals were obtained under collecting permit provided by Instituto Chico Mendes de Conservação da Biodiversidade (Sistema de Autorização e Informação em Biodiversidade #48080–2). All aspects of the study were carried out in accordance with protocols approved by the Ethics Committee on Animal Use of Instituto Butantan (protocol #1046/13).

### Anatomy, histology and histochemistry

Eight adult specimens of *Rhinella icterica*, *Rhinella marina*, *Rhinella schneideri* and *Rhinella jimi* (*n* = 2, for each species) were euthanized with a lethal dose of thiopental and skin samples from dorsal, ventral and pelvic patch and the entire macroglands of all individuals were preserved in Bouin 4% or PBS buffered paraformaldehyde (pH 7.2). After dehydration in ethanol, the samples were embedded in transversal and longitudinal orientations using paraffin and glycol methacrylate (historesin Leica®). The blocks were sectioned in a Microm® HM 340 microtome (0.5 to 6 μm thick), using disposable steel blades (for paraffin) or glass knives (for historesin). The sections were stained with haematoxylin-eosin (for paraffin) or toluidine blue-fuchsine (for historesin). For basic characterization and distribution of the chemical composition of the glands the following histochemical methods were applied: bromophenol blue (for proteins), alcian blue pH 2.5 and periodic acid-Schiff (PAS) (for acid and basic mucopolysaccharides, respectively), modified Masson-Fontana (for biogenic amines), and Sudan black B (for lipids).

### Ultrastructure

For transmission electron microscopy (TEM), fragments of dorsal warts and of the macroglands of two individuals per species were extracted and fixed according to Karnovsky (5% glutaraldehyde and 4% paraformaldehyde in 0.1 M cacodylate buffer, pH 7.2), post-fixed in 1% osmium tetroxide in 0.1 M cacodylate buffer and contrasted in 0.5% aqueous uranyl with 13.3% sucrose. After dehydration, the samples were embedded in epoxy resin (Polybed, Electron Microscopy Science). Ultrathin sections were contrasted with 2% uranyl and lead citrate and examined under the LEO 906E transmission electron microscope, operating at 80 kV.

For scanning electron microscopy (SEM), skin samples and macroglands were fixed following the same protocol described above, immersed in dimethyl sulfoxide (DMSO), frozen in liquid nitrogen and cryofractured with the aid of a frozen steel blade. After dehydration in ethanol, the samples were mounted on aluminium stubs, dried in a critical point apparatus, covered with gold in a sputtering device, and examined in a FEI Quanta 250 scanning electron microscope, operating at 10–12 kV.

### Poison extraction and dilution

Poison samples from dorsal warts, as well as macroglands, were extracted from the animals by manual compression, collected in plastic tubes and kept in a freezer at − 20 °C. After thawing, part of each sample was diluted in a solution of ultrapure water containing 0.1% trifluoroacetic acid (TFA) and 5% acetonitrile (ACN), yielding a poison solution of approximately 5 mg/mL. For analysis by mass spectrometry, the samples were resuspended in ultrapure water containing 5% acetonitrile and 0.5% formic acid. The amount of proteins present in the poison was estimated using the bicinchoninic acid method.

### Unidimensional electrophoresis (SDS-PAGE) e zymography

To analyse the protein profiles, poison samples (15 μg diluted in 30 μL) of the dorsal warts and macroglands were loaded onto a 12% polyacrylamide gel (PAGE) containing sodium dodecyl sulphate (SDS), under non-reducing conditions, according to Laemmli [44]. After separation of the proteins by electrophoresis, the gels were stained with Coomassie brilliant blue or silver. In order to evaluate the presence of proteases in the poisons, zymography was applied [36, 37, 45], using casein, gelatine and fibrinogen as substrates in the SDS- PAGE 12%. After completion of electrophoresis, the gels were processed, stained with Coomassie brilliant blue and immersed in bleach solution. Clear areas in the gel indicated where enzymatic activity occurred.

### RP-HPLC and mass spectrometry

A RP-HPLC reversed phase high performance liquid chromatography system (20A Prominence, Shimadzu Co., Japan) was used to obtain the chromatographic profile and purification of the secretions of the skin poison glands and parotoids with a C18 column (ACE C18, 5 μm, 100 Å, 250 mm × 4.6 mm). Chromatography was performed by injecting 10 μL of poison, eluted in a gradient from 0 to 100% of B in 30 min, solvent A being TFA/water (1:1000) and solvent B TFA/ACN/water (1:900:100), in a constant flow of 1 mL/min, at 38 °C. The eluted content was monitored by a Shimadzu SPD-M20A PDA detector, in the range of 200 to 500 nm.

Mass spectrometry (MS and MS^n^) were performed on-line in a nanoHPLC (UltiMate HPLC System, LC Packings, Dionex, USA) coupled to the mass spectrometer (LC-MS/MS). An ESI spectrometer (LCQDuoTM, ThermoFinnigan, USA) was used with a nanospray source. The samples were introduced into the spectrometer at a flow rate of 1 μL/min. The voltage used in the spray was 1.8 kV and the capillary voltage was 46 V, in a temperature of 180 °C. Spectra were obtained in the range of 50 to 2000 m/z. Data acquisition and processing was performed by Xcalibur.

Peaks and ions were identified according to the physicochemical characteristics and by comparison of the fragmentation spectrum reported in different studies [33, 41, 46–48] and our own database.

## Abbreviations

ACN: Acetonitrile
COM: Common poison gland
DMSO: Dimethyl sulfoxide
LC-MS/MS: Liquid chromatography–mass spectrometry
MS: Mass spectrometry
MS^n^: Sequential Mass Spectrometry
PAR: Parotoid poison gland
PAS: Periodic acid-Schiff
PER: Peripheral parotoid poison gland
RP-HPLC: Reversed-phase high-performance liquid chromatography
SDS-PAGE: Sodium dodecyl sulphate polyacrylamide gel electrophoresis
SEM: Scanning electron microscopy
TEM: Transmission electron microscopy
TFA: Trifluoroacetic acid
